# The Interfacial Dilational Rheology of Surfactant Solutions with Low Interfacial Tension

**DOI:** 10.3390/molecules30030447

**Published:** 2025-01-21

**Authors:** Guoxuan Ma, Qingtao Gong, Zhicheng Xu, Zhiqiang Jin, Lei Zhang, Guiyang Ma, Lu Zhang

**Affiliations:** 1 College of Petroleum Engineering, Liaoning Petrochemical University, Fushun 113001, China; gyvze6@163.com; 2 Key Laboratory of Photochemical Conversion and Optoelectronic Materials, Technical Institute of Physics and Chemistry, Chinese Academy of Sciences, Beijing 100190, China; gongqt@mail.ipc.ac.cn (Q.G.); zhchxu@mail.ipc.ac.cn (Z.X.); jinzhiqiang@mail.ipc.ac.cn (Z.J.); zhanglei@mail.ipc.ac.cn (L.Z.)

**Keywords:** spinning drop method, interfacial tension, interfacial dilational rheology, extended surfactant, betaine

## Abstract

In this paper, the spinning drop method was used to measure the oil–water interfacial dilational modulus of four different types of surfactants with low interfacial tension (IFT), including the anionic surfactant sodium dodecyl sulfate (SDS), the nonionic surfactant Triton X-100 (TX100), the zwitterionic surfactant alkyl sulfobetaine (ASB), and the extended surfactant alkyl polyoxypropyl ether sodium sulfate (S-C_13_PO_13_S). Based on the experimental results, we found that the spinning drop method is an effective means of measuring the interfacial dilational modulus of the oil–water interface with an IFT value of lower than 10 mN/m. For common surfactants SDS and TX100, the interfacial dilational modulus decreases rapidly to near zero with an increase in concentration when the IFT is lower than 1 mN/m. On the other hand, ASB has the highest interfacial dilatation modulus of 50 mN/m, which comes from the flatness of its unique hydrophilic group structure. The interfacial dilational modulus of S-C_13_PO_13_S showed a moderate plateau value of 30 mN/m with a broader concentration change. This is due to the fact that the main relaxation process dominating the interfacial film properties comes from the long helical polyoxypropyl chain. Through the large-size hydrophilic groups in betaine molecules and the long PO chains in the extended surfactant molecules, an interfacial film with controllable strength can be formed in a low IFT system to obtain a higher interfacial dilational modulus. This is of great significance in improving the emulsification and oil displacement of chemical flooding in reservoir pores.

## 1. Introduction

With the rapid development of the economy, the demand for crude oil is gradually increasing. It is still necessary to increase the extraction of crude oil for meeting the demand of crude oil for economic development at this stage [1]. Enhanced oil recovery (EOR) through chemical flooding provides effective assistance for sustained and stable reservoir output [2]. Surfactants have been recognized as powerful assistants in EOR from the 1970s as they can significantly reduce interfacial tension (IFT), alter the wetting properties of reservoir rocks, reduce capillary forces, and promote oil mobility, thereby enhancing oil recovery [3,4,5,6,7]. IFT is particularly important in crude oil extraction, especially the acquisition of low IFT. Therefore, the study of the surfactants behavior in low IFT or even ultra-low IFT systems is particularly important for EOR.

With the deeper exploitation of oil fields, betaines and extended surfactants show more excellent temperature and salt resistance properties than other surfactants in increasingly harsh reservoir conditions. Alkyl sulfobetaine (ASB), as a type of zwitterionic surfactant, is highly resistant to temperature and salinity due to its unique hydrophilic structure with zwitterionic groups [8]. Due to its large difference in the space occupied by hydrophilic and hydrophobic groups at the interface, betaines have the ability to synergize with other surfactants to achieve ultra-low IFT [9,10]. At the same time, ASB can resist external perturbation by the regulation of the hydrophilic portion laying upright and flat when it is subjected to external perturbation, and thus, it has a high interfacial film strength [11,12,13]. Extended surfactants are formed by inserting a moderately polar group between the hydrophobic alkyl chain and the hydrophilic ionic head of the surfactant, such as a propylene oxide (PO) or ethylene oxide (EO) group. The introduction of an appropriate amount of PO groups can not only adjust the hydrophilic–lipophilic balance (HLB) of surfactants but also increase the spatial size of hydrophobic groups on the oil side through the helical curling of PO groups on the oil side, so as to achieve the effect of “spatial matching” [14,15]. Due to the helical coiling of its flexible PO linkages on the oil phase side, intra-interfacial processes play a dominant role in the interfacial film properties when its adsorbed film is perturbed [16].

With the in-depth research related to EOR, it has been found that IFT is not the only decisive indicator of crude oil recovery, especially regarding the development of tools such as the microscopic visualization of oil displacement [17]. The strength of the interfacial film between the oil droplets and the flooding solutions also has a large impact on the crude oil displacement effect. Past experience has shown that a stronger oil–water interfacial film can reduce the tendency of oil droplets to rupture, slow emulsion breakage, and increase emulsion stability [18,19,20,21]. In recent years, studies from micro-replacements have shown that medium-strength interfacial films are more favorable for the movement of crude oil or emulsion in the pore space. Modulation of the interfacial film strength can lead to a further increase in the replacement rate compared with a lower IFT [22].

Interfacial dilational rheology is an effective means of studying the properties of interfacial adsorbed films. The drop profile analysis method used in the present experimental study can only measure the dilational rheological parameters when the IFT is above 5 mN/m. When the IFT reaches 5 mN/m or even lower, the droplet will elongate due to the decrease in IFT and finally fall. Unlike the drop profile method, the spinning drop method can be used to study interfacial dilational rheology down to 0.01 mN/m [23].

As an effective means of measuring low IFT, the spinning drop method is widely used in various industries and fields, especially in the petrochemical field. In recent years, the interfacial dilational rheology of chemical flooding with a low IFT value measured by the improved spinning drop method has become a hot topic. Zhang et al. investigated the dilational rheological properties of sodium 2-propyl-4,5-diheptylbenzene sulfonate (DHPBS) at the decane–water interface using the spinning droplet method, and the effects of experimental conditions such as oil droplet volume, rotational speed, and oscillation amplitude on the dilational modulus have been investigated [24]. Alvarado et al. performed dilational rheology experiments by dissolving C7 asphaltenes in solvents such as toluene, xylene, toluene/xylene mixtures (50/50 bulk phase), and decanal proteins and screened surfactant–oil–water (SOW) systems based on the lowest value of dilational modulus versus phase angle for SOWs with hydrophilic–lipophilic equilibrium (HLDN) values of zero [25]. Marquez et al. investigated the interfacial properties of the nonionic surfactants NPEO6 and NPEO8 and the extended surfactant C_13_PO_14_EO_2_SO_4_ solutions with cyclohexane, kerosene, and heavy crude oil, making a preliminary study of the experimental conditions and a preliminary comparison with the drop profile analysis method [26]. In addition, they found that the measured interfacial dilatation modulus and phase angle of the SOW system were at a minimum at HLDN = 0, providing a novel screening tool for the optimal formulation screening of the SOW system [26,27,28]. They used this method to measure the interfacial elastic and viscous moduli between the anionic surfactant sodium dodecylbenzene sulfonate (SDBS) and the co-surfactants sec-butanol solution and cyclohexane. It was found that the minimum values of interfacial elastic and viscous moduli occurred at the optimal hydrophilic–oleophilic equilibrium oil–water system, which was related to the rapid exchange of surfactant between oil and water [29]. They measured the modulus and phase angle between sodium dodecyl sulfate (SDS), n-pentanol, and kerosene and found that the very low modulus and phase angle values are at HLDN = 0, which are believed to be related to the presence of a bi-continuous phase micro-emulsion as well as to the rapid surfactant exchange between the native body and the interface [30]. They also present the effects of salts on the interfacial performance of a SDBS–heptane system at optimum formulation, i.e., hydrophilic–lipophilic deviation (HLD) = 0 [31].

So far, the influence of surfactant structure on the interfacial dilational modulus under low IFT is still less studied. In this paper, four different types of surfactants were selected for the measurements of the oil–water interfacial dilational modulus, including the anionic surfactant sodium dodecyl sulfate (SDS), the nonionic surfactant tralatone X-100 (TX100), the zwitterionic surfactant alkyl sulfobetaine (ASB), and the extended surfactant sodium alkyl polyoxypropylene ether sulfate (S-C_13_PO_13_S). Due to the particularity of the molecular structure, ASB and S-C_13_PO_13_S exhibit higher interfacial moduli under high interfacial tension conditions. SDS and TX100 are representatives of common surfactants [12,16]. The results of this study are helpful to understand the interfacial behavior of surfactants with high interfacial activity.

## 2. Results and Discussion

### 2.1. Effect of Surfactant Concentration on IFT

**Figure 1:**
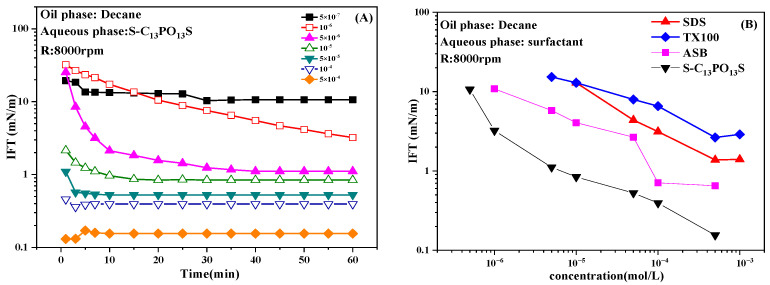
Dynamic IFTs of S-C_13_PO_13_S solutions (**A**) and equilibrium IFTs of SDS, TX100, ASB, and S-C_13_PO_13_S solutions (**B**) at different concentrations against decane.

Figure 1 shows the dynamic IFTs of S-C_13_PO_13_S and equilibrium IFT values of SDS, TX100, ASB, and S-C_13_PO_13_S solutions against decane. In Figure 1, the IFT decreases as the surfactant concentration increases. With the passage of time, the IFT gradually reaches equilibrium, while among the four surfactants, the extended surfactant has a stronger ability to reduce the interfacial tension.

As the surfactant concentration increases, the adsorption of the surfactant at the oil–water interface increases and the IFT decreases continuously. Meanwhile, the decrease in IFT depends not only on the hydrophilic and lipophilic equilibrium of the surfactant but also on the sizes of hydrophilic and hydrophobic parts of molecules on both sides of the interface. The decrease in IFT results from the adsorption of the surfactant at the interface, and the generation of an ultra-low interfacial tension requires that there be as few solvent molecules (water and oil molecules) as possible in the interfacial layer. This requires not only a sufficient number of surfactant molecules at the interface but also a suitable size of surfactant molecules to facilitate the formation of a dense adsorption film. If the hydrophilic and hydrophobic parts of the surfactant molecules occupy the same space on both sides of the interface, it can strongly reduce the IFT.

For extended surfactants, the area occupied by the hydrophobic side is determined by the helically coiled PO chains. It was found that with the increase in surfactant adsorption at the oil–water interface, the whole PO chain in the oil was helically erected in the form of a thin cylinder, which achieved the effect of lateral superposition and longitudinal stretching in the oil phase side, resulting in a better spatial match with the hydrophilic side [32]. With the increase in the number of PO chains, the curled PO chains occupy more space at the interface and match the size of the anionic head, and when their number reaches 13, ultra-low IFT values may appear [33]. Thus, among the four surfactants, it has the ability to reduce the IFT to a lower value.

### 2.2. Effect of Rotational Speed and Rotational Speed Amplitude Magnitude on Interfacial Dilational Modulus Measurements

**Figure 2:**
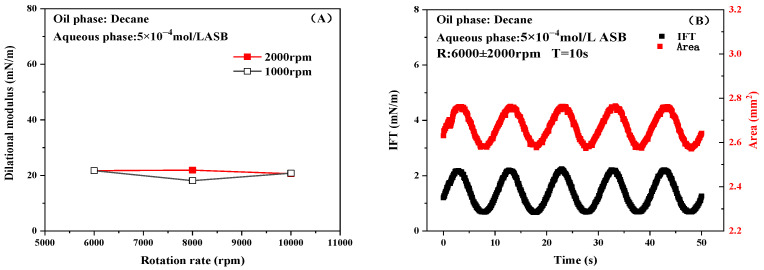

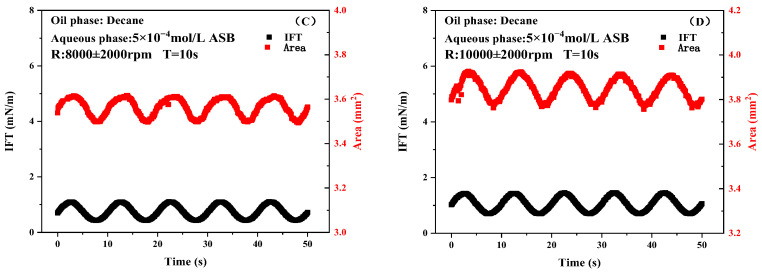
Interfacial dilational modulus at 6000, 8000, and 10,000 rpm and at an amplitude magnitude of 1000 and 2000 rpm (**A**) and changes in area and IFT at 6000 (**B**), 8000 (**C**), and 10,000 (**D**) rpm with an amplitude magnitude of 2000 rpm of 5 × 10^−4^ mol/L ASB solution.

The rotational speed employed to measure the IFT and the amplitude magnitudes of the sinusoidal periodic oscillation of rotational speed to disturb the interface are two decisive factors for measuring dilational rheology. Figure 2 shows the interfacial dilational modulus of 5 × 10^−4^ mol/L ASB solution at 6000, 8000, and 10,000 rpm and at an amplitude magnitude of 1000 or 2000 rpm. As shown in Figure 2, it can be clearly observed that the response of the interface to the change in rotational speed, the change in IFT versus area, exhibits a clear sinusoidal pattern when the rotational speed cycle is oscillated. Therefore, it can be confirmed that the measurement of the interfacial dilational modulus by the rotating drop method is an effective means of carrying out the dilational rheology study of liquid–liquid interfaces with low IFT values. In addition, the interfacial area gradually increased with the increase in rotational speed, and the change of rotational speed and amplitude magnitude did not make the measurement of interfacial dilational modulus change significantly. Therefore, within a certain range of rotational speed and rotational speed amplitude, the rotational speed and rotational speed amplitude do not affect the calculation of the interfacial dilational modulus. The measurement of the interfacial dilational modulus using the spinning drop method is a more reliable experimental tool.

In order to obtain a wider measurement range under the limited experimental conditions, the base rotational speed of 8000 rpm and rotational speed amplitude of 2000 rpm were selected for the following experiments.

### 2.3. Effect of Frequency on Interfacial Dilational Modulus

**Figure 3:**
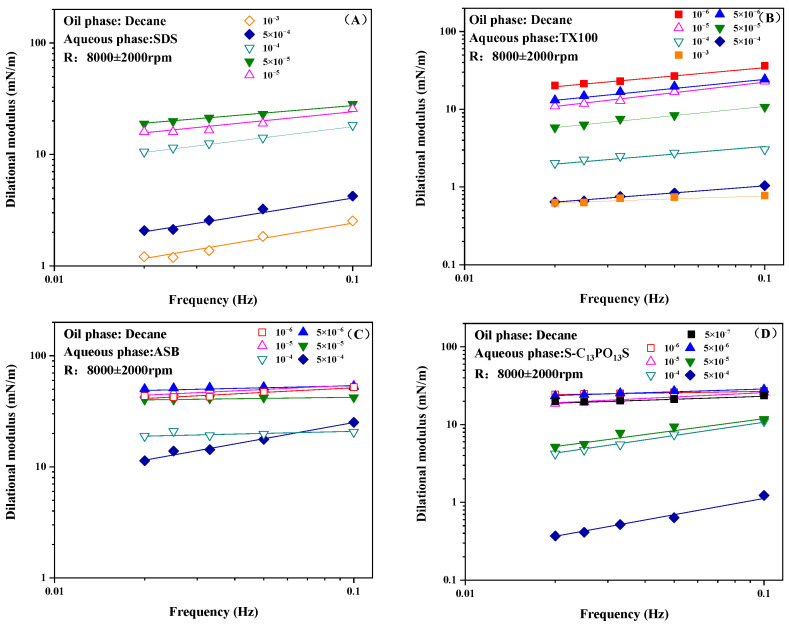
Influence of frequency on the dilational modulus for SDS (**A**), TX100 (**B**), ASB (**C**), and S-C_13_PO_13_S (**D**).

For the surfactant adsorption film with various relaxation processes, the working frequency will affect the interfacial rheological parameters, thus reflecting the interfacial behavior of the adsorbed molecules. Figure 3 shows the influence of frequency on the dilational modulus for the SDS, TX100, ASB, and S-C_13_PO_13_S solutions. The dilational modulus was measured in the range of 0.02 to 0.1 Hz to investigate the frequency dependence of the interfacial modulus.

The interfacial dilational modulus of the surfactant at the decane–water interface increases gradually with oscillation frequency. At much lower frequencies, the surfactant molecules have enough time to reduce the IFT gradient caused by the interfacial deformation. As the oscillation frequency increases, the recovery of the interfacial tension becomes slower compared with the rapid change in interfacial area, resulting in a higher IFT gradient. As a result, the interfacial dilational modulus increases with increasing oscillation frequency [34]. Generally speaking, the lgε-lgω curve is usually quasi-linear for surfactant solutions, suggesting that the characteristic frequency of the relaxation process at or near the interfacial layer may be higher than the highest oscillation frequency (0.1 Hz) used in this experiment [35]. According to the literature, the slopes of the lgε-lgω curves are different when there are different relaxation processes in the system. Different relaxation processes in the system will lead to different adsorption film properties [36]. Therefore, the slope data of the lgε-lgω line will quantitatively represent the viscoelasticity of the adsorption film.

**Figure 4:**
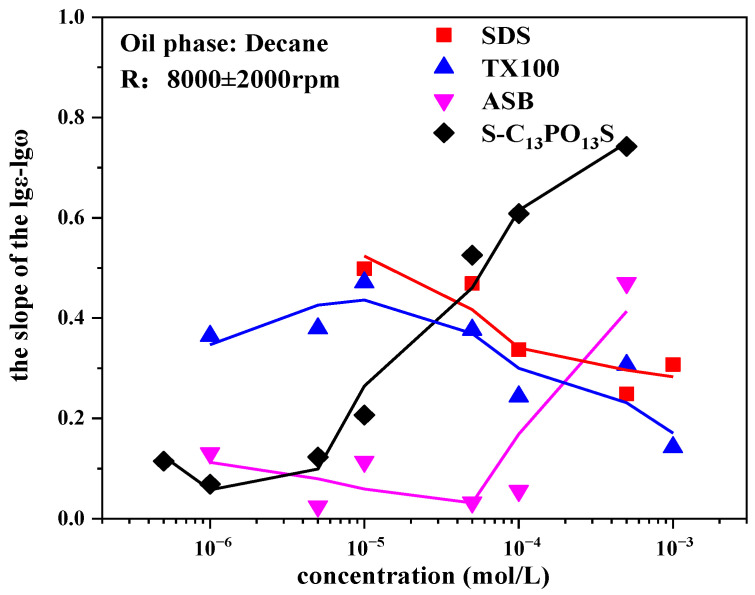
Influence of concentration on the slope of the lgε-lgω on SDS, TX100, ASB, and S-C_13_PO_13_S.

Figure 4 shows the influence of concentration on the slope of the lgε-lgω of SDS, TX100, ASB, and S-C_13_PO_13_S solutions. As shown in Figure 4, there is a large difference between different surfactants in the experimental frequency range. It is well known that the smaller the slope, the more elastic the adsorption film [12]. For common surfactants, such as SDS and TX100, when the interfacial film is periodically disturbed, the interactions between molecules at the interface are weak, and the molecular exchange between the interface and the bulk phase dominates the nature of the interfacial film, and the slopes of the lgε-lgω line do not vary much with concentration, with values generally being around 0.2–0.5.

For the zwitterionic surfactant ASB, the slopes are smaller over a wide range of concentrations, with values being maintained below 0.2, due to the fact that when the interfacial film is perturbed, the in-film relaxation process in which the unique structure of the hydrophilic side is flattened in an upright position greatly increases the elasticity of the adsorbed interfacial film [11,12]. At higher bulk phase concentrations, the molecular exchange between the bulk phase and the interface increases with the increase in bulk phase concentration, resulting in a decrease in the elasticity of the adsorbed film and an increase in the slope value.

For the extended surfactant S-C_13_PO_13_S, the helically coiled flexible PO chains on the hydrophobic side also suffer from the in-film relaxation phenomenon of upright lying flat when the interfacial film is perturbed, and thus, the elasticity of the interfacial film is higher at the initial concentration, with a slope value of around 0.1 [16]. However, due to the longer PO groups and alkyl chains on the hydrophobic side, the space occupied by them fits better with the space of the hydrophilic groups, so the adsorption increases significantly with the concentration, making the dynamic relaxation process at the interface the main factor dominating the interfacial viscoelasticity; therefore, the decrease in the elasticity of the interfacial adsorption film and the slope increases. Similarly, at higher concentrations, the increase in diffusive exchange causes the slope to increase further.

### 2.4. Effect of Surfactant Concentration on Interfacial Dilational Modulus

**Figure 5:**
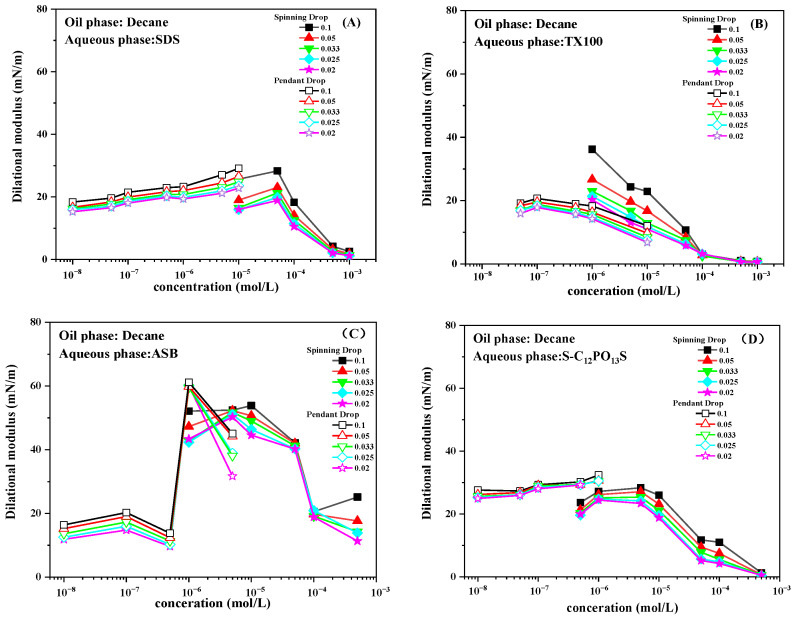
Influence of bulk concentration on the dilational modulus for SDS (**A**), TX100 (**B**), ASB (**C**), and S-C_13_PO_13_S (**D**).

Figure 5 shows the variation of the interfacial dilational modulus with the concentration of the four surfactants measured by a combination of the drop shape analysis method and the spinning drop method. It can be seen that the interfacial dilational modulus of all four surfactants passed through a maximum as a function of bulk concentration, which is consistent with the general law of surfactants. Meanwhile, from the perspective of the change rule of the interfacial dilational modulus, there is no big difference in the numerical calculation results between the two surfactants, although they adopt different measurement means and calculation methods. On the other hand, the drop profile analysis method is more suitable for the measurement of high IFT systems, while the spinning drop method is more suitable for low tension and even ultra-low IFT systems. Especially when the tension is less than 10 mN/m or even lower, the spinning drop method can be competent at this time, as well as the task of quantitatively characterizing the ability of adsorption film to resist interface deformation during the screening of the formulation of the chemical flooding. 

**Figure 6:**
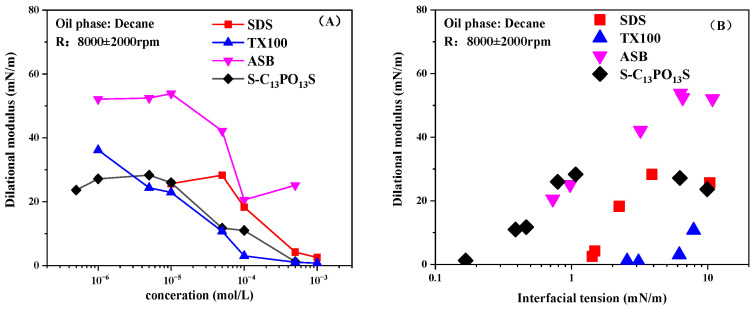
Influence of bulk concentration (**A**) and IFT (**B**) on the dilational modulus for SDS, TX100, SB, and S-C_13_PO_13_S solutions.

Figure 6 shows the variation of the interfacial dilational modulus with concentration and IFT for the four types of surfactants at a frequency of 0.1 Hz. In general, the interfacial dilational modulus continues to decrease as the interfacial tension decreases when the magnitude of IFT reaches single digits [37]. Meanwhile, the interfacial dilational modulus of surfactants is generally a non-monotonic function of both surfactant concentration and IFT [38].

As shown in the figure, ASB has the highest interfacial dilatation modulus compared with the other three surfactants, reaching 50 mN/m for concentrations below 10^−4^ mol/L. This is due to its unique hydrophilic group structure, which makes it so that when interface compression is performed, the upright flatness of the hydrophilic group at the interface can greatly improve the interfacial film strength, and the relaxation process of molecules within the interface is dominant, resulting in a higher interfacial dilatation modulus [16].

The extended surfactant, S-C_13_PO_13_S, has a moderate interfacial dilational modulus over a wide range of concentrations and IFTs compared with other surfactants. As the adsorption continues to increase, the SDS modulus gradually decreases to almost zero when the IFT is low below 5 mN/m. However, compared with SDS without PO chains, the interfacial dilational modulus of S-C_13_PO_13_S remained constant at IFT tensions of 1–10 mN/m, with moduli of around 20 mN/m. It can be hypothesized that this is due to the introduction of PO chains, which lead to a plateau value in the higher measurement range. However, unlike the entanglement of EO groups on the aqueous side [39], PO groups increase the interfacial dilational modulus by increasing the difficulty of the dynamic relaxation process of surfactant molecules within the interface, such as the rearrangement of interface molecules as well as molecular orientation. Therefore, the main factor controlling the performance of the interfacial film is no longer the diffusive exchange with the bulk phase but the dynamic relaxation process of the molecules at the interface, and thus, a large plateau value of the interfacial dilational modulus exists. As the concentration increases again, at higher concentrations, the increase in bulk phase concentration leads to increased molecular exchange between the bulk phase and the interface, resulting in a weakening of the interfacial film strength and a decrease in the dilatation modulus.

In a word, through the large-size hydrophilic groups in betaine molecules and the long PO chains in the extended surfactant molecules, an interfacial film with controllable strength can be formed in a low IFT system to obtain a higher interfacial dilational modulus. This is of great significance to improve the emulsification and oil displacement of chemical flooding in reservoir pores.

## 3. Materials and Methods

### 3.1. Materials

**Figure 7:**
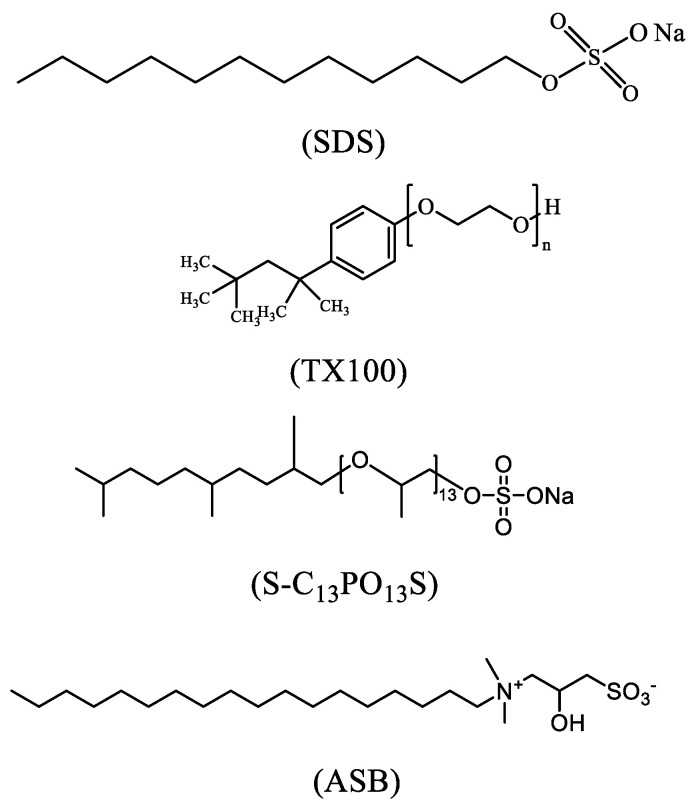
The structure of SDS, TX100, ASB, and S-C_13_PO_13_S.

The surfactants used in this paper, sodium dodecyl sulfate, SDS (critical micelle concentration (CMC), 1.9 × 10^−2^ mol/L); and Triton X-100 (CMC, 2.9 × 10^−4^ mol/L), were purchased from Xilong Chemical Co (Shantou, China) with a purity of more than 95%. Alkyl sulfobetaine, ASB (CMC, 2.2 × 10^−6^ mol/L), was synthesized in our laboratory with a purity of more than 95%, and alkyl sulfate, S-C13PO13S (CMC, 1.2 × 10^−5^ mol/L), was purchased from Sasol Chemical Co. (Sandton Houston, TX, USA), with a purity of more than 95%. The structures and abbreviations of the surfactants are shown in Figure 7. The decane used in the interfacial tension experiments was of analytical purity and was purchased from Aladdin Biochemical Technology Co. (Shanghai, China). The inorganic reagents used were of analytical grade. All surfactant solutions were prepared using double-distilled water (resistivity > 18.2 MΩ·cm) by adding 1.0% NaCl.

### 3.2. Measurements of Interfacial Tension and Interfacial Dilational Rheology

#### 3.2.1. Spinning Drop Method

A CNGTX spinning drop interfacial dilational rheometer (Beijing Shengwei Technology Co., Ltd., Beijing, China) was used to measure the IFT as well as the interfacial dilational modulus of surfactant solutions with low IFT values. The surfactant solution was injected into the glass tube as the external phase, and decane was injected into the center of the glass tube as the internal phase. The sample was considered to be in equilibrium when the measured value of IFT was kept constant for 30 min. The experimental temperature was controlled at 25.0 ± 0.5 °C. The measurement error of the IFT values was less than 5% [40].

After the IFT was balanced, the rotational speed was changed sinusoidally. The rotational speed amplitude was set to 1000 or 2000 rpm, and the oscillation periods were 10 s, 20 s, 30 s, 40 s, and 50 s, respectively, thus achieving the purpose of periodic perturbation of the droplet. In the course of the cyclic perturbation, the HD camera of the interfacial rheometer autonomously tracked the droplets and maintained the level. Thereafter, the recorded video captured the interfacial tension and interface area during the ensuing change process. In the end, we obtain the dilational modulus by fitting the sinusoidal curves and calculating them.

When the interface is subjected to periodic compression and expansion, the interfacial tension changes periodically, and the interfacial dilational modulus is defined as the ratio of the change in interfacial tension to the change in relative interfacial area [41].

$$\varepsilon =\frac{d\gamma }{d\ln A}$$

where ε is the interfacial dilational modulus, γ is the IFT, and A is the interfacial area.

#### 3.2.2. Drop Shape Analysis Method

The measurements of the interface dilational rheology by drop shape analysis method were carried out using an LSA100OEDM optical dilational rheometer (Beijing Eastern Dataphy Instrument Co., Ltd., Beijing, China). The details of the experiment are shown in the literature [16].

## 4. Conclusions

In this work, the interfacial dilational rheological properties of SDS, TX100, ASB, and S-C_13_PO_13_S solutions were studied. Based on the experimental results, the following conclusions can be drawn:

(1) During the measurements of the interfacial dilational modulus with a low IFT value by the spinning drop method, the influence of the rotational speed magnitude and amplitude of the sinusoidal periodic oscillation of rotational speed on the measurement process was investigated. A base rotational speed of 8000 rpm and sinusoidal periodic oscillation amplitude of 2000 rpm were proved to be appropriate.
(2) At low bulk concentration, the in-interface relaxation process of ASB and S-C_13_PO_13_S determines the properties of the adsorption film, and the elasticity of the adsorption film is larger. At high concentration, a variety of relaxation processes contribute greatly to the viscosity.
(3) The modulus of conventional surfactants such as SDS and Triton X-100 decreases to near zero when the interfacial tension is lower than 1 mN/m. However, through the large-size hydrophilic groups in betaine molecules and the long PO chains in the extended surfactant molecules, an interfacial film with controllable strength can be formed in a low IFT system to obtain a higher interfacial dilational modulus. This is of great significance to improve the emulsification and oil displacement of chemical flooding in reservoir pores.

## Data Availability

Data are contained within the article. Complete contact information is available at: http://datadryad.org/stash/share/Vyu8GiLMJO4V0Z0sdEK4gL0swSDCEGDbZr0lamGK6r4 (accessed on 3 January 2025).
